# Comparison of the efficacy of small-incision clamp-assisted reduction and open reduction for the treatment of femoral shaft fractures with an anterograde intramedullary nail: a retrospective study

**DOI:** 10.1186/s13018-022-03067-8

**Published:** 2022-03-28

**Authors:** Shufeng Lin, Zefeng Zhang, Yipeng Yan, Yizhong Li, Jinkuang Lin, Hui Ye

**Affiliations:** The Second Affiliated Hospital of Fujian Medical University, 34 Zhongshan North Road, Licheng District, Quanzhou City, Fujian Province China

**Keywords:** Femoral shaft fractures, Small incision clamp reduction, Open reduction, Intramedullary nail

## Abstract

**Background:**

To compare the efficacy of small-incision clamp-assisted reduction with open reduction for the treatment of femoral shaft fractures by anterograde intramedullary nailing.

**Methods:**

The data of 63 patients with femoral shaft fractures, treated between January 2016 and June 2021, were retrospectively analyzed. All patients received anterograde intramedullary nail fixation, and the OA/OTA classification of fractures was 32-C. The average follow-up period was 13 months (range: 11–14 months). According to the method of fracture reduction, patients were divided into a small-incision clamp-reduction group (referred to as the clamp-reduction group) and an open-reduction group. The reduction time, operative time, the number of fluoroscopy, intraoperative blood loss, postoperative VAS score, postoperative time to discharge, and the rates of intraoperative and postoperative complications were compared between the two groups.

**Results:**

There were statistically significant differences in reduction time, operative time, the number of fluoroscopy, intraoperative blood loss, postoperative VAS score, postoperative time to discharge (*t* = 6.718, − 11.679, 18.963, − 11.609, − 22.432, − 7.187; *P* < 0.05). In the clamp-reduction group, there was no intraoperative blood transfusion. However, there were one case of wound infection and one case of deep vein thrombosis after operation. In the open-reduction group, ten patients received intraoperative blood transfusion, one patient developed hemorrhagic shock, two patients developed wound infection, and two patients developed bone nonunion during follow-up.

**Conclusions:**

Both groups had good functional recovery after operation. However, compared with open reduction, clamp reduction is a safer reduction method with shorter operation time, less intraoperative blood loss, less postoperative pain, shorter hospital stay and fewer postoperative complications.

## Background

Femoral shaft fractures refer to a tubular bone fracture between 2 and 5 cm below the smaller trochanter and 2–4 cm above the femoral condyle, which account for approximately 4–6% of all fractures and are mostly caused by high energy trauma [1, 2]. These fractures mostly occur in adults aged from 20 to 40 years. As the numbers of traffic accidents increase, the proportion of adult incidence is on the rise, with a male-to-female ratio of approximately 2.8:1 [3]. Currently, closed reduction and interlocking intramedullary nail fixation is a standard treatment for femoral shaft fracture [4, 5]. While influenced by thigh muscle strength and complexity of the fracture, closed reduction often involves problems such as difficulty in reduction, long reduction time, poor fracture combination, and a potential increase in the radiation risk to both doctor and patient [6], especially in the treatment of femoral multiple fracture. Georgiadis et al. [7] reported that the fracture reduction time could be shortened by fixing both ends of the fracture with a Schanz nail to support the femoral shaft fracture. However, a Schanz nail is relatively thick in diameter, which hinders the passage of the guide wire and destroys the bone cortex and the soft tissue of the surrounding muscles. Ma et al. [8] proposed a method of reduction by clamping the fracture end with a bone holder clamp. However, the 3–5-cm surgical incision required for this method results in greater damage to the tissue and a risk of damage to blood vessels and nerves. Consequently, we developed an improved version of the two methods above by clamping the middle and the distal end of the fracture with toothed vascular forceps and assisting the guide wire through the medullary cavity, which required a smaller incision and caused less tissue damage. Data of femoral fracture from January 2016 to June 2021 were analyzed retrospectively in this study in order to compare the efficacy of our small-incision clamp-assisted reduction with that of open reduction for the treatment of femoral shaft fractures with anterograde intramedullary nailing and to clarify the advantages of small-incision clamp reduction.

## Methods

### Patient inclusion and exclusion criteria

Inclusion criteria were as follows: (1) patients with femoral shaft fracture that the OA/OTA classification of fractures was 32-C according to imaging examination; (2) fracture was treated surgically by small-incision clamp-assisted reduction or open reduction with anterograde intramedullary nailing.

Exclusion criteria were as follows: (1) patients with open or pathological fractures; (2) patients with bilateral femoral shaft fractures; (3) patients suffering from circulatory diseases and on long-term anticoagulation drugs; (4) fracture combined with injuries or bleeding in other tissues and organs. All patients provided written informed consent.

### Research objective and evaluation criteria

The data of 63 patients with femoral shaft fractures, treated between January 2016 and June 2021, were analyzed. All patients received anterograde intramedullary nail fixation, and the OA/OTA classification of fractures was 32-C. According to the method of fracture reduction, patients were divided into a small-incision clamp-reduction group (referred to as the clamp-reduction group) and an open-reduction group. There were 17 males and 14 females in the clamp-reduction group, with an average age of 54.26 ± 18.95 years, while in the open-reduction group there were 19 males and 13 females, with an average age of 50.94 ± 19.11 years. The average follow-up period was 13 months (range: 11–14 months).

The reduction time, operative time, number of fluoroscopies, intraoperative blood loss, postoperative VAS score, postoperative time to discharge, and the rates of intraoperative and postoperative complications were compared between the two groups. Evaluation criteria were as follows: (1) reduction time: The time the guide wire entered the distal end of the femoral medullary cavity from the moment the reduction began to the time when it was reset satisfactorily; (2) operation time: The time required from when the operation began until the surgical wound suture and dressing was completed; (3) fluoroscopy times: refers to the total number of fluoroscopies using a “C-arm” X-ray fluoroscopy machine during the operation; (4) intraoperative blood loss: evaluated by the increase in the net weight of gauze (with 1 g considered to equal 1 mL) and drainage fluid in the negative pressure suction tank (minus flushing fluid) [9]. Electronic weigher was be used to measure during the operation. (5) Postoperative VAS score: on the first day after the surgery, patient pain was assessed using a numerical evaluation scale where 0–3 was mild pain, 4–6 was moderate pain, and 7–10 was severe pain. (6) Postoperative to discharge time: the number of days after the operation ended until the patient was discharged. (7) Intraoperative and postoperative complications. (8) Hospital for Special Surgery score of the knee joint: The score is evaluated from pain (30 points), function (22 points), range of motion (18 points), muscle strength (10 points), fixed deformity (10 points), instability (10 points), etc. The higher the score, the better the function.

### Surgical method

**Fig. 1:**
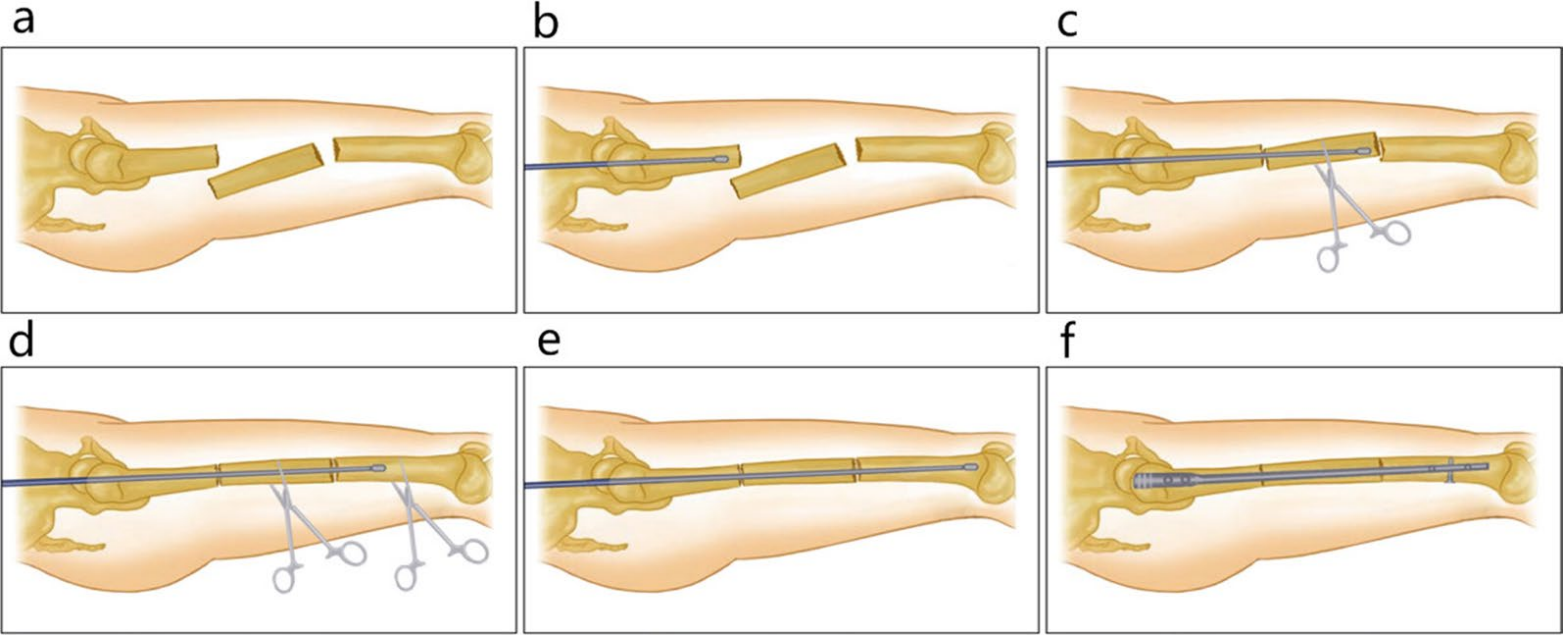
A small-incision clamp-reduction schematic. **a** A lateral imaging of the femur shows a three-segment fracture. **b** The guide wire was inserted to the proximal end of the fracture. **c**, **d** The toothed vascular forceps were inserted, and the direction and angle of the toothed vascular forceps were adjusted to push the guide wire to the distal fracture. **e**, **f** An intramedullary nail after reaming

After combined spinal–epidural anesthesia, the patient was placed in a supine position on the traction bed, the affected limb was fixed in a neutral position, the unaffected leg was fixed on the traction frame in a scissor position to avoid affecting intraoperative fluoroscopy, and the position of the fracture end was identified and marked on the body surface. The surgical site was routinely disinfected and covered with sterile drapes, and a small incision of approximately 3 cm was created near the proximal femoral intertrochanter and the femoral long axis. After opening the skin, subcutaneous tissue and tensor fascia lata, followed by longitudinal dissociation of the gluteus medius and gluteus minimus, the greater trochanter was exposed, and the guide wire was inserted with the help of a “C-arm” X-ray fluoroscopy machine. A pulp chamber bur was used to open the cortex along with the guide wire, then a “golden finger” was pushed along the hole into the proximal end of the fracture, and the olive-tipped guide wire was inserted to the proximal end of the fracture (Fig. 1b). An incision was made in the skin at the outer aspect of the thigh or anterior lateral middle fracture block under fluoroscopy, with an incision of approximately 0.5 cm; the toothed vascular forceps were inserted along the incision, the middle fracture block was clamped and the direction and angle of the toothed vascular forceps were adjusted to push the guide wire to the middle fracture (Fig. 1c). A second incision of 0.5 cm was made in the skin outside the distal fracture block, the toothed vascular forceps were inserted, and the direction and angle of the toothed vascular forceps were adjusted (Fig. 1d) to push the guide wire to the distal fracture. The length was then measured after capturing a satisfactory position under fluoroscopy, the pulp cavity was reamed, an intramedullary nail was inserted, screws were locked, and the tail cap was installed (Fig. 1f). Finally, after capturing a satisfactory position under fluoroscopy, the wound was washed with physiological saline and stitched after stopping the bleeding. A cocktail of antibiotics was given intravenously 30 min before and after surgery to prevent infection.

### Postoperative management

**Fig. 2:**
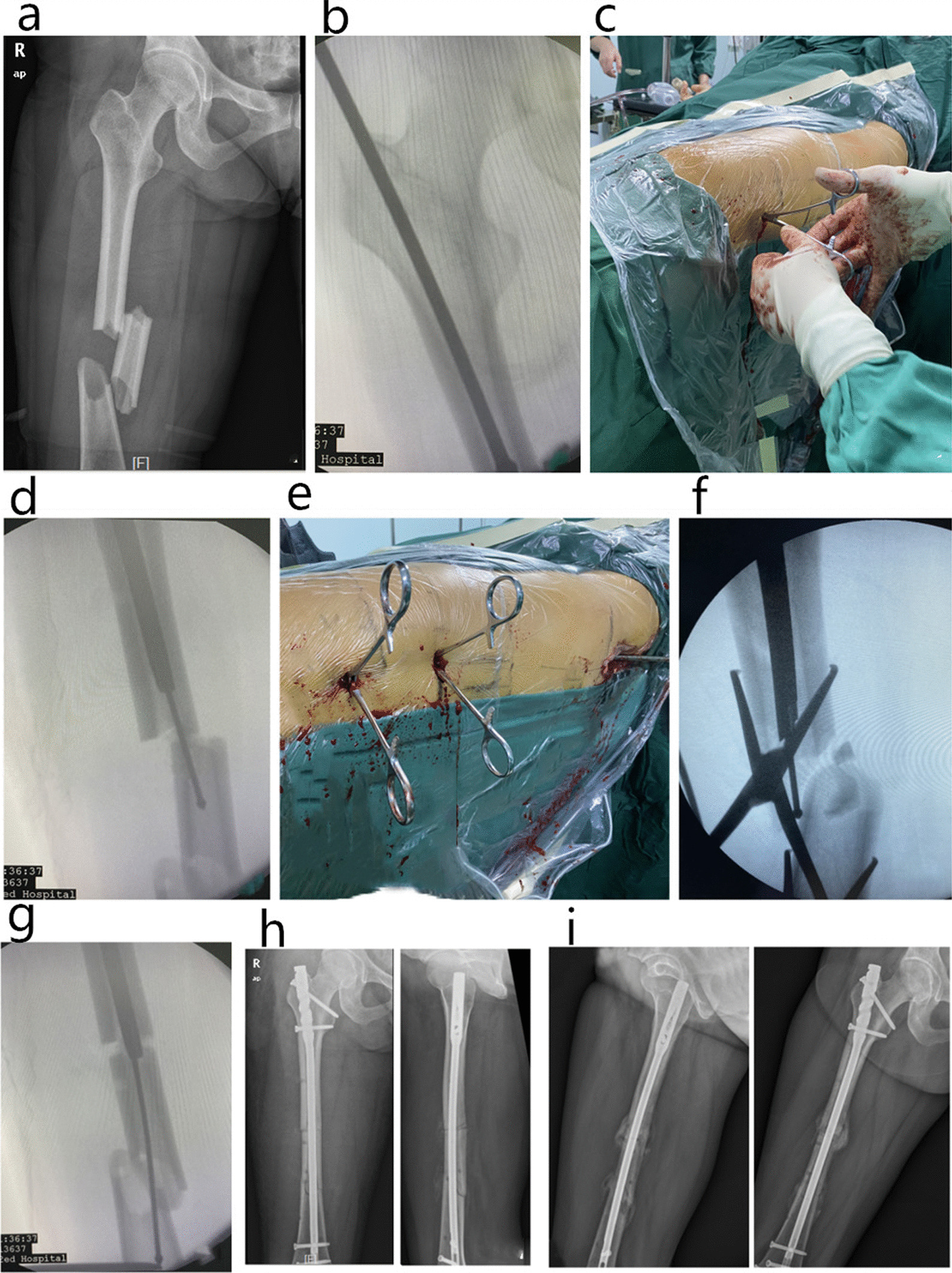
A 35-year-old female patient with a multi-segment femoral shaft fracture caused by a traffic accident. **a** Preoperative X-ray shows that the AO type of femoral shaft fracture was a 32-C fracture. **b** The intramedullary nailing point was ascertained and the guide wire was inserted; **c** An incision of 0.5 cm was cut in the skin by the lateral side of the medial femoral fracture block and toothed vascular forceps were inserted. **d** The intermediate fracture block was clamped using the toothed vascular forceps, and the guide wire was pushed by controlling the direction of the fracture block. **e–g** In the same way, the toothed vascular forceps were inserted into the lateral side of the fracture block at the distal end of the femur, and the guide wire was inserted, while the direction of the fracture block at the middle and the distal end was controlled. **h** An intramedullary nail after reaming. **i** Radiographs at 6 months postoperatively

After recovery from anesthesia, active analgesic treatment was administered and regular anteroposterior and lateral femoral radiographs were taken. A cocktail of antibiotics was given intravenously within 24 h after surgery to prevent infection. Twenty-four hours after the operation, the patient was given low-molecular-weight heparin anticoagulant therapy, and at the same time, the patient was advised to start muscle tension contraction of the quadriceps femoris, gastrocnemius, soleus and other muscles to prevent the development of deep vein thrombosis in the lower limbs. Regular X-ray examinations were taken monthly for half a year after the operation to observe callus formation, and weight bearing was gradually increased according to the progress of fracture healing (Fig. 2).

### Statistical methods

Statistical software SPSS20.0 was used for all statistical analyses. The normally distributed measurement data were expressed as *X* ± *S*. Student’s *t*-test was used for comparison between two groups, and analysis of variance was used for comparison among multiple groups. Differences in count data between groups were compared by *χ*^2^ test, and *P* < 0.05 was considered statistically significant.

## Results

### General results

The OA/OTA classification of femoral shaft fractures in the 63 patients in both groups was 32-C fracture, and all patients underwent operation as planned with successful insertion of intramedullary nails. There were 31 patients in the clamp-reduction group, comprising 17 males and 14 females, with an average age of 54.26 ± 18.95 years. In the open-reduction group, there were 32 patients, comprising 19 males and 13 females, with an average age of 50.94 ± 19.11 years. There was no statistically significant difference in gender or age between the two groups (*P* > 0.05), indicating comparability. For the comparison of general data between the two groups, see Table 1.

**Table 1:** Comparison of general data between the clamp-reduction group and the open-reduction group

Group	Cases	Gender (case)		Age (Y)	Injured side		Time from injury to operation (d)
		Male	Female		Left	Right	
Clamp-reduction group	31	17	14	54.26 ± 18.95	13	18	5.4 ± 1.6
Open-reduction group	32	19	13	50.94 ± 19.11	15	17	5.2 ± 1.8
Statistic		*χ*^2^ = 0.132		*T* = 0.692	*χ*^2^ = 0.156		*T* = 0.393
*P*-value		0.716		0.491	0.693		0.696

### Evaluation index results

The fracture reduction time and the number of intraoperative fluoroscopy in the clamp-reduction group were significantly higher than those in the open-reduction group, with increases of 37.8% and 179.9%, respectively. The differences between the two groups were statistically significant (*P* < 0.05, see Table 2 for details). The operative time, intraoperative blood loss, postoperative VAS score and postoperative time to discharge in the clamp-reduction group were significantly lower than those in the open-reduction group by 18.9%, 74.53%, 38.0% and 46.5%, respectively, and the differences between the two groups were statistically significant (*P* < 0.05, see Table 2 for details).In the clamp-reduction group, none of the patients required blood transfusion during the operation, but there was one case of wound infection and one case of lower-extremity deep vein thrombosis after operation. In the open-reduction group, ten patients received blood transfusion during the operation, and one patient developed hemorrhagic shock, two patients developed wound infection, and two patients developed bone nonunion during follow-up. The HSS score of the clamp-reduction was significantly higher than those in the open-reduction group on the first day, the first month and the third month after surgery (*P* < 0.05). However, there was no significant difference between the two groups on the sixth and twelfth month after surgery (*P* > 0.05, see Table 3 for details).

**Table 2:** Comparison of evaluation indexes between the clamp-reduction group and the open-reduction group

Group	Cases	Fracture reduction time (min)	Operative time (min)	Times of intraoperative fluoroscopy (time)	Intraoperative blood loss (mL)	Postoperative VAS score (point)	Postoperative to discharge time (d)
Clamp reduction group	31	28.03 ± 4.25	97.52 ± 8.56	12.68 ± 2.27	158.39 ± 72.16	4.61 ± 0.49	6.0 ± 1.67
Open reduction group	32	20.34 ± 4.81	120.31 ± 6.79	4.53 ± 0.76	621.88 ± 213.62	7.44 ± 0.50	11.22 ± 3.74
*T*-value	–	6.718	− 11.679	18.963	− 11.609	− 22.438	− 7.187
*P*	–	< 0.05	< 0.05	< 0.05	< 0.05	< 0.05	< 0.05

**Table 3:** The HSS knee function score between the clamp-reduction group and the open-reduction group after surgery

Group	Case	The first day after surgery	The first month after surgery	The third month after surgery	The sixth month after surgery	The twelfth month after surgery
Clamp reduction group	31	46.90 ± 2.71	59.13 ± 2.74	81.84 ± 3.27	88.87 ± 2.32	96.84 ± 1.27
Open reduction group	32	29.78 ± 2.81	46.63 ± 2.04	75.63 ± 1.66	89.69 ± 1.67	96.50 ± 1.14
*T*-value	–	24.577	20.474	9.470	− 1.606	1.118
*P*	–	< 0.05	< 0.05	< 0.05	> 0.05	> 0.05

## Discussion

The results of this study showed that there were no significant differences in gender, age, injury side or time from injury to operation between the clamp-reduction group and the open-reduction group (*P* > 0.05). The reduction time of fracture (28.03 ± 4.25 min) and the number of intraoperative fluoroscopies (12.68 ± 2.27) in the clamp-reduction group were higher than those in the open-reduction group (20.34 ± 4.81 min and 4.53 ± 0.76, respectively) and the differences were statistically significant (*P* < 0.05). The operative time (97.52 ± 8.56 min), intraoperative blood loss (158.39 ± 72.16 mL), postoperative VAS score (4.61 ± 0.49) and postoperative time to discharge (6.0 ± 1.67 d) in the clamp-reduction group were significantly lower than those in the open-reduction group (120.31 ± 6.79 min, 621.88 ± 213.62 mL, 7.44 ± 0.50, and 11.22 ± 3.74 d, respectively, and the differences were statistically significant (*P* < 0.05). These data show that small-incision clamp-assisted reduction is quick and effective, shortening the operation time, reducing postoperative blood loss, relieving postoperative pain and shortening hospitalization time. In addition, the incidences of intraoperative blood transfusion and postoperative complications in the clamp-reduction group were lower than those in the open-reduction group, which increased patient safety. In our study, there was significant difference between the two groups on the first day, the first month and the third month after surgery (*P* < 0.05). However, there was no significant difference on the sixth and twelfth month after surgery (*P* > 0.05). This shows that the clamp-reduction group is better than the open-reduction group in knee joint function within the first few months after the surgery. With the increase in functional exercise, there was no difference in knee function between the two groups.

In clinical practice, the method of closed reduction and internal fixation with an intramedullary nail under fluoroscopy is generally adopted for the treatment of adult femoral shaft multi-segment fractures [10]. However, as the whole femoral shaft is surrounded by strong muscle groups, different fracture segments are pulled by different muscles, resulting in angulation deformity, which makes closed reduction very difficult [11]. In recent years, some surgeons have adopted open reduction, limited small-incision reduction and Schanz nail-assisted reduction to reduce femoral shaft fracture. Open reduction has the advantages of short fracture-reduction time and the need for fewer episodes of intraoperative fluoroscopy. However open reduction requires stripping of more muscle causing greater trauma, which leads to delayed fracture healing or nonunion and increased intraoperative and postoperative blood loss. The infection rate after open reduction and intramedullary nail fixation of femoral fracture incision has been reported to be 10%, while it is only 1% after closed reduction [12]. This study further supports the above contention. The limited small-incision reduction is a frequently adopted method of inserting an interlocking intramedullary nail at present. Although it minimizes the destruction of soft tissue and periosteum at the fracture end, it usually requires a 2–4 cm surgical incision and still damages soft tissues such as muscle [13]. Compared with the previous two methods of femoral shaft fracture reduction, the Schanz method of nail-assisted reduction causes less trauma, but the diameter of Schanz nails are relatively thick, thus causing a relatively large amount of damage to the bone cortex and easily destroying the surrounding muscle and soft tissue [14]. For multi-segment femoral shaft fractures, the main difficulties of the operation are to pass the guide wire through the fracture end and to control postoperative rotational deformity [15, 16]. Consequently, we adopted a small incision and used the clamp technique with toothed vascular forceps to assist reduction and internal fixation of the interlocking intramedullary nail in the treatment of such fractures, and the clinical curative effect was satisfactory. During the operation, the skin was cut in the middle of the lateral or anterolateral thigh and the distal fracture block under fluoroscopy, and the toothed vascular forceps were inserted along the incision, with the direction of the fracture block controlled by the vascular forceps to penetrate the guide wire.

Small-incision clamp-assisted reduction has the following advantages: (1) The intraoperative reduction incision is small, at approximately 0.5–1 cm, and it therefore causes less damage to soft tissue, significantly shortens the surgical incision closure time, reduces intraoperative blood loss, relieves postoperative pain, shortens the hospitalization time, and advances the recovery process. (2) The intermediate fracture block is clamped directly by the toothed vascular forceps, and the force acts directly on the femur, which reduces the difficulty of operation and makes it easier to control the rotation of the intermediate fracture block. (3) Toothed vascular forceps are a commonly used orthopedic instrument which is convenient for sampling regardless of special conditions and region. However, there are points requiring attention in the clinical practice of small-incision clamp-assisted reduction to reduce adult femoral multi-segment fracture: (1) This technique should not be used for patients with thigh vascular and nerve injury to avoid exacerbating vascular and nerve injury during reduction. (2) Before intraoperative reduction, a traction bed or other traction tools should be used to correct the shortening of the femur, and avoid blind reduction that aggravates femoral injury. (3) This technique is not suitable for patients with severe osteoporosis or severe comminuted or pathological fractures of the femur. (4) The technique increases the number of fluoroscopies and the vital parts of the patient's body need to be protected from radiation exposure.

There are some limitations in this study. First, this study is a retrospective study with a relatively small sample size. The data could be biased, so a larger sample size, or even multi-center studies, is needed for verification. Secondly, the fracture type in this study was purely 32-C fracture, yet the distribution of the fracture line was different and the muscle attached to different fracture pieces also differed, which could result in partial deviation during the operation as well as other factors.

## Conclusion

In summary, small-incision clamp-assisted reduction and open reduction for the treatment of femoral shaft fractures with anterograde intramedullary nailing both have a good curative effect, but small-incision clamp-assisted reduction is a safer reduction method with the advantages of shorter operation time, less intraoperative blood loss, lighter postoperative pain, shorter hospitalization time and fewer postoperative complications.

## Data Availability

The datasets used and/or analyzed during the current study are available from the corresponding author on reasonable request.
